# Targeting the stimulator of interferon genes (STING) in breast cancer

**DOI:** 10.3389/fphar.2023.1199152

**Published:** 2023-06-28

**Authors:** Ma Ying-Rui, Bai Bu-Fan, Liu Deng, Shi Rong, Zhou Qian-Mei

**Affiliations:** ^1^ Institute of Interdisciplinary Integrative Medicine Research, Shanghai University of Traditional Chinese Medicine, Shanghai, China; ^2^ Shuguang Hospital Affiliated to Shanghai University of Traditional Chinese Medicine, Shanghai, China; ^3^ Shanghai Institute of Stem Cell Research and Clinical Translation, Shanghai, China

**Keywords:** STING, DNA damage response, tumour immune microenvironment, mitochondrial function, STING agonists

## Abstract

Breast cancer has a high occurrence rate globally and its treatment has demonstrated clinical efficacy with the use of systemic chemotherapy and immune checkpoint blockade. Insufficient cytotoxic T lymphocyte infiltration and the accumulation of immunosuppressive cells within tumours are the primary factors responsible for the inadequate clinical effectiveness of breast cancer treatment. The stimulator of interferon genes (STING) represents a pivotal protein in the innate immune response. Upon activation, STING triggers the activation and enhancement of innate and adaptive immune functions, resulting in therapeutic benefits for malignant tumours. The STING signalling pathway in breast cancer is influenced by various factors such as deoxyribonucleic acid damage response, tumour immune microenvironment, and mitochondrial function. The use of STING agonists is gaining momentum in breast cancer research. This review provides a comprehensive overview of the cyclic guanosine monophosphate-adenosine monophosphate synthase-STING pathway, its agonists, and the latest findings related to their application in breast cancer.

## 1 Introduction

Breast cancer is a prevalent malignancy and ranks as the second leading cause of mortality among women, following lung cancer (Sung et al., 2021; Siegel et al., 2022; Xia et al., 2022). It poses a significant risk to women’s health and negatively affects their quality of life. The increasing incidence and mortality rates associated with this disease exert a substantial financial burden on the healthcare industry, highlighting the need for innovative and effective therapeutic interventions. Current treatment modalities for breast cancer include surgery, radiotherapy, chemotherapy, and endocrine therapy, with paclitaxel, platinum, anthracyclines, and capecitabine being the primary chemotherapy agents used (Kerr et al., 2022). Immunotherapy has also emerged as a valuable approach for treating breast cancer, as it yields improved treatment outcomes (Emens, 2018).

The cyclic guanosine monophosphate (GMP)-adenosine monophosphate (AMP) synthase (cGAS) and stimulator of interferon genes (STING) (cGAS-STING) signalling pathway has become a prominent subject of interest in cancer immunotherapy. This pathway detects deoxyribonucleic acid (DNA) in the cytoplasm and stimulates the production of immune factors such as type I interferon (IFN) (IFN-I), initiating a cascade of immune responses with an anti-tumour effect (Deng et al., 2014). Moreover, it enhances the immune response when paclitaxel and platinum-based agents are administered in oncological chemotherapy (Della Corte et al., 2020; Hu Y. et al., 2021; Cao et al., 2022). Particularly, paclitaxel has been closely associated with the activation of the cGAS-STING pathway in breast cancer treatment (Zhu C. et al., 2022; Qiu et al., 2022), suggesting that targeting this pathway offers a novel approach for treating breast cancer. This review focuses on the cGAS-STING pathway and its current implications in DNA repair, the tumour microenvironment (TME), and mitochondrial function in breast cancer. Additionally, it examines existing therapies and emerging targets for treating this disease.

## 2 The cGAS-STING pathway

Innate immunity serves as the first line of defence against foreign genetic material and plays a crucial role in tumour-induced immune responses. The primary DNA sensor that triggers the innate immune response is cGAS, a protein found throughout the cell that plays crucial roles in anti-tumour immunity, autophagy, cellular senescence, defence against microbial infection, and autoimmune and inflammatory diseases (Zhang et al., 2020; Zhang et al., 2021). Upon DNA recognition, cGAS activates STING protein, which detects cyclic dinucleotides from the endoplasmic reticulum (ER) membrane (Zhu Z. et al., 2022). The cGAS-STING signalling pathway comprises three main phases: double-stranded DNA detection, intracellular signalling, and immune response activation (Yu et al., 2022). Through these stages, the cGAS-STING pathway becomes a central link between immunity and cancer. These details are discussed below.

The DNA sensor cGAS, belonging to the nucleotidyl transferase family, catalyses 2′3′-cyclic GMP-AMP (cGAMP) formation. cGAMP induces conformational changes and oligomerisation of the STING protein. As a result, the activated STING forms tetramers and translocates from the ER to the Golgi apparatus (Dobbs et al., 2015). Subsequently, the activated protein recruits and activates TANK-binding kinase 1 (TBK1) and IFN regulatory factor 3 (IRF3), resulting in the expression of various antiviral genes (Zhang et al., 2020). Among these genes are IFNs-I, which significantly influence the therapeutic efficacy of several anti-cancer drugs, including immunotherapies (Civril et al., 2013; Sun et al., 2013; Zitvogel et al., 2015; Zhou et al., 2020). IFN-α and IFN-β are the most common type I IFNs, promoting the activation and proliferation of cytotoxic T lymphocytes, natural killer (NK) cells, dendritic cells (DCs), and B cells, thereby bridging the innate and acquired immune response (Zhou et al., 2022). The STING protein also activates components of the nuclear factor kappa B (NF-κB) signalling pathway; however, the specific activation mechanism remains unresolved (Burdette et al., 2011; Hopfner and Hornung, 2020). Furthermore, the cGAS-STING signalling pathway induces an anti-tumour immune response by sensing the DNA damage response (DDR) and stimulating the innate immune response within tumours (Jiang et al., 2021).

The STING gene is expressed in various cell types, and studies focused on tumours, it is frequently observed that STING signalling is suppressed. The inhibition is attributed to loss-of-function mutations or epigenetic silencing of the cGAS/STING promoter region (Konno et al., 2018). Apart from transcriptional regulation, different types of modifications could also affect the function of STING. The primary forms of modifications include polyubiquitination and phosphorylation, followed by palmitoylation, sumoylation, oxidation, nitro-alkylation, carbonylation, and disulphide bond formation (Zhang et al., 2022a). Within the cGAS-STING pathway, STING translocation from the ER to the Golgi apparatus is crucial for the activation of the STING signalling pathway. Additionally, it has been reported that the binding of the ER calcium sensor stromal interaction molecule 1 to STING specifically retains STING in the ER, preventing its translocation to the Golgi apparatus. This in turn prevents STING activation and blocks the STING cascade response (Guerini, 2022). Furthermore, the presence of a key factor that terminates STING signalling at the Golgi apparatus, namely, articulin complex 1, facilitates the sorting of phosphorylated STING into lattice-protein-coated transport vesicles for delivery to the endolysosomal system, resulting in its degradation and the termination of STING-dependent immune activation (Liu Y. et al., 2022).

Targeting the cGAS-STING pathway is an emerging therapeutic approach for various cancers due to compelling evidence indicating its activation in the TME elicits potent anti-cancer effects. However, it is crucial to note that the activated cGAS-STING pathway might also exhibit pro-cancer functions under specific conditions. For instance, the cGAS-STING-mediated IFN-I response and the IFN-I-associated senescence could promote tumour initiation by producing various protumourigenic cytokines (Boukhaled et al., 2021). Additionally, the STING protein induces interleukin (IL)-35 production, which activates regulatory B cell functions but simultaneously inhibits NK cell responses (Li et al., 2022). These contrasting roles suggest that the STING pathway operates through diverse mechanisms that require thorough investigation to develop effective targeted therapies.

## 3 DDR and the cGAS-STING pathway in breast cancer

Genomic instability is a prominent feature that promotes the malignant transformation of cancer (Alhmoud et al., 2020). Defects in DDR and increased replication stress are critical events that promote the clonal evolution of cancer cells by promoting genetic alterations such as gene copy number changes, chromosomal rearrangements, and gene mutations, thereby facilitating tumour progression (Pilié et al., 2019). Notably, DDR deficiency plays a pivotal role in determining tumour immunogenicity, and growing evidence support the notion that DDR-targeted therapy increases anti-tumour immune response (Chabanon et al., 2021). Additionally, cytotoxic drugs that target the DDR pathway are employed as anti-cancer therapies, as this pathway governs many mechanisms underlying tumour cell resistance and sensitivity to cytotoxic radiotherapy (Jiang et al., 2021). For instance, cisplatin, a first-line chemotherapeutic agent for various malignant tumours including breast (Zhu Y. et al., 2022), ovarian (Yang et al., 2022), head and neck (Kwon et al., 2021), lung (Fennell et al., 2016), and bladder (Herr and Soloway, 2022) cancers, exerts its anti-cancer effects by inducing DDR in cancer cells (Dasari and Tchounwou, 2014; Tchounwou et al., 2021).

cGAS activation can occur through two distinct mechanisms: the accumulation of DNA in the cytoplasm and micronuclei or prolonged auto-activation of the DDR signalling (Ragu et al., 2020). For instance, cGAS detects fragmented DNA produced by DDR, leading to changes in cGAMP, activating the cGAS-STING pathway and initiating an immune response (Harding et al., 2017; Reisländer et al., 2020). Consequently, DNA released into the cytoplasm after a DDR event serves as a crucial cGAS trigger, and the absence of cGAS decreases DDR signalling (Banerjee et al., 2021). Blocking DNA replication and repair affects genome integrity and activates the cGAS-STING signalling cascade (Chabanon et al., 2019). Moreover, mutations in DDR-related genes, which impair their function, result in increased expression of genes associated with the cGAS-STING pathway in non-small cell lung cancer (Della Corte et al., 2022). Similarly, pharmacological inhibition of polyadenosine diphosphate-ribose polymerase (PARP) and checkpoint kinase 1 (CHK1) in small-cell lung cancer results in the inhibition of the DDR pathway and activates the cGAS-STING pathway, evoking an anti-tumour immune response (Sen et al., 2019). These findings suggest the need for further investigation into the mechanisms by which DDR activates the cGAS-STING pathway.

Patients with breast cancer often exhibit alterations in DDR genes. For instance, approximately 10.7% of female patients carry deleterious mutations in cancer susceptibility genes, with 6.1% attributed to breast cancer gene (BRCA) 1/2% and 4.6% involving other susceptibility genes such as checkpoint kinase 2, ataxia-telangiectasia mutated (ATM), BRCA1 interacting helicase 1, partner and localiser of BRCA2, phosphatase and tensin homolog, nibrin, RAD51C, RAD51D, mutS homolog 6, and PMS1 homolog 2, mismatch repair system component (Tung et al., 2016). BRCA1 and BRCA2, in particular, play a crucial role in homologous recombination-mediated DNA repair (Krishnan et al., 2021). Germline defects in these genes can contribute to DDR dysfunction in breast cancer, thereby activating the cGAS/STING signalling pathway and eliciting an immune response (Parkes et al., 2017). Therefore, inhibiting DNA repair and promoting DDR progression, which activates the cGAS-STING pathway, present promising avenues for cancer therapy.

DDR-targeted therapies are emerging as promising strategies for treating breast cancer, particularly triple-negative breast cancer, wherein overexpression of DNA repair proteins, such as PARP1 and replication protein A, might alter the sensitivity to chemotherapy and DDR inhibitors (Lee et al., 2020). For instance, IFI16 has demonstrated anti-tumour effects in triple-negative breast cancer by inducing STING-mediated IFN-I production (Ka et al., 2021; Huang et al., 2022). However, the DDR-induced cGAS-STING-mediated IL-6 -signal transducer and activator of transcription 3 pathway in triple-negative breast cancer has been associated with reduced patient survival (Vasiyani et al., 2022). These contrasting findings suggest that the DDR-induced cGAS-STING signalling pathway plays a bidirectional regulatory role in breast cancer, highlighting the importance of investigating the mechanisms that control its directionality in this disease.

Therefore, inhibiting DNA repair and promoting DDR progression, which activates the cGAS-STING pathway, represents a promising avenue for cancer therapy. Other drugs, such as PARP1 inhibitors, have already gained approval for treating breast and ovarian cancers, demonstrating remarkable efficacy (Staniszewska et al., 2022; Tian et al., 2022). Similarly, inhibitors targeting DDR-related genes, such as DNA-dependent protein kinase, catalytic subunit, ATM, ataxia telangiectasia and Rad3-related protein, CHK1, and WEE1, exhibit promising anti-cancer effects (Wengner et al., 2020). Furthermore, paclitaxel activates cGAS by affecting cell mitosis, inducing cGAS-STING pathway-dependent IFN-I responses (Hu Y. et al., 2021).

## 4 Tumour immune microenvironment and the cGAS-STING pathway in breast cancer

The TME encompasses various components, including the vasculature, extracellular matrix, and non-carcinoma cells, which play crucial roles in tumour initiation, progression, invasion, and metastasis (Bahrami et al., 2018). Significantly, the TME has garnered substantial attention in cancer therapy research due to the potential anti-cancer effects associated with activating the cGAS-STING pathway in the TME (Li and Bakhoum, 2022). In breast cancer, targeting the TME holds great promise and has demonstrated excellent therapeutic outcomes (Mehraj et al., 2021; Zheng et al., 2022). The TME in breast cancer exhibits variable cellular composition and structural characteristics, serving as a central regulator of tumour progression (Danenberg et al., 2022). Immune-activating cells within the TME include tumour-infiltrating lymphocytes, NK cells, and dendritic cells, while immune-suppressing cells comprise T regulatory cells, tumour-associated macrophages, and myeloid-derived suppressor cells. The breast cancer stroma comprises cancer-associated fibroblasts, vascular endothelial cells, and mesenchymal stromal cells (Wilson et al., 2022). Targeting specific cells within the TME, such as eosinophils, tumour-associated macrophages, cancer-associated fibroblasts, tumour-infiltrating lymphocytes, and regulatory CD^4+^/CD^8+^ T cells, can enhance anti-tumour immunity in breast cancer (Li Y. et al., 2020; Li D. et al., 2020; Wu et al., 2020; Grisaru-Tal et al., 2021; Soongsathitanon et al., 2021; Sun et al., 2021). Targeting tumour-associated macrophages has demonstrated significant alleviation of chemotherapy resistance in breast cancer.

Evidence suggests that elevated levels of tumour-infiltrating lymphocytes within the TME play a crucial role in treating breast cancer (Ahn et al., 2020). Differential analysis of tumour compartments has revealed that patients with triple-negative breast cancer responsive to chemotherapy exhibit high STING protein levels (Kulasinghe et al., 2021), indicating its presence in the TME of breast cancer and its potential as a treatment target. While chimeric antigen receptor T (CAR-T) cells are a type of cell treatments for treating haematological malignancies, their effectiveness against solid tumours is limited (Tchou et al., 2017). However, when the cGAS-STING pathway is activated within the breast cancer TME, T helper/IL-17-producing CD8^+^ T -generated CAR-T cells show increased persistence in the TME and enhanced tumour control (Xu et al., 2021). The STING protein induces the production of IFN-β by intra-tumoural DCs, which initiates and recruits T cells into the TME (Foote et al., 2017). Nanoparticles loaded with STING agonists activate the cGAS-STING signalling pathway within the TME, resulting in IFN-β production and the activation of antigen-presenting cells, thereby stimulating the activation of tumour-reactive cytotoxic T cells (Covarrubias et al., 2022). Consequently, STING agonists hold significant promise as a therapy for reshaping the immunosuppressive TME (Wehbe et al., 2021), as they can reverse its immunosuppressive nature and sensitise breast cancer to immunotherapy (Chen et al., 2020; Zhang L. et al., 2022; Shen et al., 2022).

## 5 Mitochondrial function and the cGAS-STING pathway in breast cancer

Mitochondrial and nuclear DNA leaking into the cytoplasm activate the cGAS-STING signalling in addition to foreign DNA (Hopfner and Hornung, 2020). Due to its location in the ER, particularly in the ER-mitochondria-associated membrane, STING protein has an inherent advantage in detecting mitochondrial stress responses (Smith, 2020). Mitochondria serve as bioenergetic, biosynthetic, and signalling organelles with crucial roles in regulating innate and adaptive immunity (Weinberg et al., 2015), particularly in processes that lead to apoptosis. In mitochondria-mediated apoptosis, activating pro-apoptotic proteins B-cell leukaemia/lymphoma 2 protein (Bcl-2) antagonist killer 1 (BAK)/Bcl-2-associated X protein (BAX) results in mitochondrial outer membrane permeabilisation, thereby inducing caspase activation and cell death (Lohard et al., 2020). Since mitochondria possess their DNA, BAK/BAX-mediated mitochondrial damage triggers the release of mitochondrial DNA (mtDNA). Consequently, the cGAS-STING-mediated cytoplasmic DNA sensing pathway identifies mtDNA and initiates apoptosis (White et al., 2014; McArthur et al., 2018; Chang S. et al., 2022). Mitochondrial inner membrane permeabilisation enables the release of mtDNA into the cytoplasm and activates the cGAS-STING signalling pathway (Riley et al., 2018).

Targeting the cGAS-STING-associated mitochondrial apoptotic pathway is emerging as a novel therapeutic approach for breast cancer. For instance, cyclic di-AMP (c-di-AMP), an analogue of cGAMP, activates the cGAS-STING pathway and induces mitochondria-mediated apoptosis in oestrogen receptor-negative breast cancer cells (Vasiyani et al., 2021). Eribulin, a microtubule-targeting agent, promotes cGAS-STING signalling expression in triple-negative breast cancer cells by facilitating the cytoplasmic accumulation of mtDNA, IFN-β production, and downstream interferon-stimulated genes (Fermaintt et al., 2021). ATM inhibition enhances the effectiveness of immune checkpoint blockade treatment in breast cancer by facilitating the cytoplasmic leakage of mtDNA and the activation of the cGAS-STING pathway (Hu M. et al., 2021).

Mitochondrial reactive oxygen species (ROS) are a vital source of endogenous ROS. In malignant cells, mitochondria exhibit ROS overproduction, which could promote cancer development by altering gene expression and participating in signalling pathways (Yang et al., 2016; Zhao et al., 2016). Thus, ROS has emerged as a target for anti-tumour therapy. In colorectal cancer, SUMO-specific proteinase 3 detects oxidative stress and promotes the STING-mediated DC-initiated anti-tumour immune response (Hu Z. et al., 2021). Mitochondrial lon, a chaperone protein, induces ROS, which could lead to mtDNA damage, activate IFN signalling via the cGAS-STING-TBK1 axis, and promote programmed death ligand 1 -mediated immune escape (Cheng et al., 2020). Sinularin differentially upregulates ROS and causes oxidative DNA damage in breast cancer cells, potentially activating the cGAS-STING pathway (Huang et al., 2018). STING protein could also act as an upstream regulator of ROS and influence the transcriptional program of ROS metabolism (Hayman et al., 2021). However, the understanding of the relationship between ROS and the cGAS-STING pathway in breast cancer is limited, and further investigation is necessary to uncover new treatment options.

## 6 Anti-cancer effects of STING agonists

Given the significant potential of the cGAS-STING signalling pathway in anti-tumour therapy, the development of STING agonists has received considerable attention. One prominent drug used as a STING agonist in preclinical studies is 5, 6-dimethylxanthenone-4-acetic acid (DMXAA, ASA404, Vadimezan), a flavonoid compound. Initially employed as a tumour vascular disruptor for anti-cancer treatment, DMXAA was later found to activate the cGAS-STING signalling in mouse models (Baguley, 2003; Daei Farshchi Adli et al., 2018). In combination with specific cancer treatment drugs, such as paclitaxel, it has demonstrated favourable efficacy in patients with intermediate to advanced non-small-cell lung cancer (McKeage et al., 2009). However, when combined with others, such as platinum-based drugs, it has demonstrated negligible effect on the outcomes of patients with triple-negative breast cancer (Lara et al., 2011). Despite its considerable potential in mouse models, DMXAA has proven unsuccessful in human clinical studies, possibly due to its inability to induce the STING signalling pathway in humans (Conlon et al., 2013). Indeed, molecular dynamics simulations revealed that dynamic structural differences between human and mouse STING proteins cause differential sensitivity to DMXAA (Shih et al., 2018). While DMXAA has demonstrated promising performance in mouse tumour models, it has laid a foundation for synthesising new derivatives that hold greater promise for cancer treatment (Hou et al., 2020; Gobbi et al., 2021).

Cyclic dinucleotides, including cGAMP and bacterial messengers c-di-AMP and cyclic di-GMP (c-di-GMP), represent valuable STING agonists (Wang et al., 2020). These compounds serve as natural ligands for the STING protein and play a crucial role in activating STING protein after cGAS-mediated cytoplasmic DNA recognition (Li and Chen, 2018). They exhibit high potential in cancer therapy. For instance, c-di-AMP induces breast cancer cell apoptosis via the cGAS-STING pathway activation and regulation (Vasiyani et al., 2021). Moreover, in a mouse model of bladder cancer, *bacillus* Calmette–Guérin overexpressing c-di-AMP improves anti-tumour effects through a STING-dependent pathway (Singh et al., 2022). Nanoparticles co-synthesised with c-di-AMP and the immunomodulatory trace element manganese significantly improved the therapeutic efficacy of STING-mediated combined radioimmunotherapy (Wang et al., 2022). Furthermore, c-di-GMP-activated STING demonstrates promising immunotherapeutic efficacy in breast cancer (Chandra et al., 2014), whereas c-di-GMP-loaded peptide nanotubes enhance immunotherapy for melanoma (Zhang et al., 2022c). As an endogenous member of the cGAS-STING signalling pathway, cGAMP exhibits significant potential as a STING agonist in anti-tumour therapy, as CAR-T cells generated using cGAMP display enhanced anti-tumour capacity in breast cancer (Xu et al., 2021; Su et al., 2022). Similarly, cGAMP enhances the anti-tumour activity of CAR-NK cells in pancreatic cancer (Da et al., 2022). These findings suggest that the effects observed with these natural agonists are dependent on the cGAS-STING pathway activation.

Clinical drug development of synthetic cyclic dinucleotides as STING agonists is underway. For instance, ADU-S100 (MIW815) activates the cGAS-STING pathway and demonstrates good tolerability in patients with advanced/metastatic solid tumours or lymphomas (Meric-Bernstam et al., 2022a). Combined treatment with ADU-S100 and the programmed cell death protein 1 (PD-1) inhibitor spartalizumab also shows a favourable safety profile in patients with advanced/metastatic solid tumours or lymphoma (Meric-Bernstam et al., 2022b). Additionally, ADU-S100 in combination with PD-1/cyclooxygenase-2 blockade suppresses peritoneal dissemination of colon cancer and elicits durable tumour immunity in colon cancer (Lee et al., 2021). Another synthetic STING agonist, MK-1454, demonstrates potent anti-tumour activity in pre-clinical trials and is currently in clinical development, showing encouraging efficacy (Harrington et al., 2018; Chang W. et al., 2022). Several other synthetic agonists, including SB11285, BMS-986301, MK-2118, GSK3745417, E7766, SNX281, SYNB 1891, TAK-676, and BI-STING, are undergoing clinical trials and studies (Challa et al., 2017; Amouzegar et al., 2021). Furthermore, several synthetic agonists such as ML-RR-S2-cGAMP, ML-RR-S2-CDG, 3′3′-cyclic AIMP, GSK532, and JNJ-4412, although not yet in the clinical research stage, hold promise for the field of cancer treatment (Corrales et al., 2015; Thomsen et al., 2020; Amouzegar et al., 2021).

In addition to the aforementioned STING agonists, there is a subset of drugs that could be used for cancer therapy by activating the cGAS-STING pathway. For instance, E7766 is a macrocyclic bridging STING agonist with high anti-tumour activity in a mouse model of liver metastases and is also considered a clinical candidate (Kim et al., 2021). Moreover, MSA-2 is a compound that binds human and mouse STING proteins, and when combined with anti-PD-1 antibodies, it inhibits tumour growth and improves survival rates (Pan et al., 2020). A novel STING agonist, MSA-1, activates STING proteins in humans and mice and can be combined with PD-1-binding inhibitors to improve anti-PD-1 resistance (Perera et al., 2022). Several other drugs, including SR-717, amidobenzimidazole, STACT-TREX1, and MV-626, are currently under investigation as STING agonists (Chin et al., 2020; Jiang et al., 2020; Liu X. et al., 2022).

## 7 Conclusion

**FIGURE 1:**
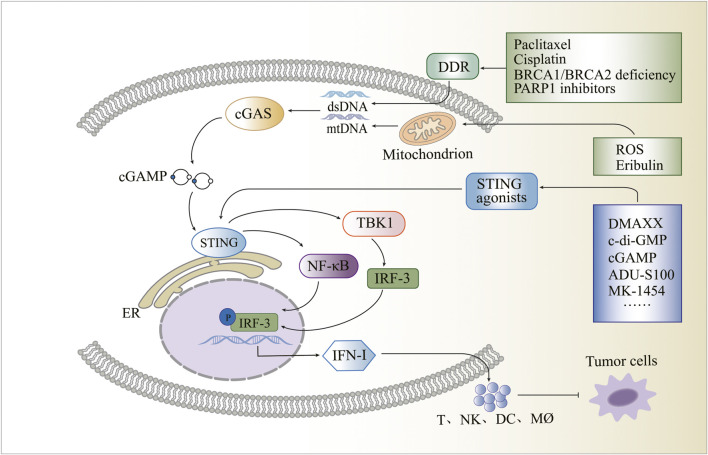
Schematic overview of the cyclic guanosine monophosphate-adenosine monophosphate synthase and stimulator of interferon genes pathway and its influencing factors.

Breast cancer poses a significant threat to women’s lives; however, the activation of the cGAS-STING pathway, which triggers an immune response, offers promising prospects for its treatment. Within the cytoplasm, the cGAS protein detects DNA and activates the STING protein, resulting in the activation of TBK1, IRF3, IFN-I, and NF-κB to produce a series of immune responses. STING activation is closely associated with DDR, and breast cancer often exhibits DDR-related gene alterations, particularly in BRCA1 or BRCA2 genes. Targeted DDR therapy has emerged as a potential therapeutic approach for breast cancer, with PARP-1 inhibitors serving as an approved example. The TME controls tumour progression, and breast cancer TME exhibits distinct characteristics. The presence of the STING protein in the breast cancer TME has been observed, influencing tumour progression. Consequently, activating the protein within the TME presents an opportunity to reshape its immunosuppressive nature. Mitochondria, which possess their DNA, are essential organelles that can activate the cGAS-STING pathway under certain conditions. Targeting the cGAS-STING-associated mitochondrial apoptotic pathway and mitochondrial ROS provides a novel avenue for breast cancer treatment. Given the promising potential of the activated cGAS-STING signalling pathway in anti-tumour therapy, various STING agonists are being developed as anti-cancer drugs. Examples include DMXAA, c-di-AMP, c-di-GMP, cGAMP, ADU-S100, MK-1454, SB11285, BMS-986301, E7766, MSA-1, and MSA-2 (Figure 1).

The activation of the STING signalling pathway serves as an innate immune sensing mechanism that results in IFN-I production within the TME. This stimulation activates immune cells within the TME, initiating an anti-tumour immune response. Consequently, STING agonists hold great promise as immunotherapeutic drugs. However, STING agonists do not exhibit inhibitory effects on all types of tumours. Currently, the STING signalling pathway is over-activated in tumours with low antigenicity, tumours that release a significant amount of DNA from the cytoplasm due to exposure to potent carcinogens, and tumours with chromosomal instability (CIN) phenotype, which might promote tumour growth and metastasis. Therefore, when considering the use of STING agonists in clinical settings, it is crucial to take into account factors such as the tumour type, antigenicity, and inflammatory microenvironment. It is necessary to understand the CIN status of the tumour and the STING basic activation level.

Understanding the molecular mechanism of STING agonists, as well as identifying and screening tumour-predictive biomarkers suitable for predicting the response to STING agonists, are critical aspects in elucidating the therapeutic potential of these agents. Additionally, selecting appropriate tumour types and optimising treatment dosages are essential for enhancing the efficacy of STING agonists while minimising adverse reactions. Therefore, the development of clinical applications for STING agonists holds great promise. The side effects and indications of STING agonists need to be further confirmed to ensure the safety and efficacy of the treatment. This can be achieved through the development of novel drug delivery systems, which can be combined with other anti-tumour therapies, such as radiotherapy, chemotherapy, targeted therapy, and immunotherapy. While there might be challenges in the research and development of drugs targeting the cGAS-STING pathway, further research on the molecular mechanisms underlying the upstream and downstream pathways and the development of drug delivery systems will pave the way for new targets for anti-tumour research.

Research into the cGAS-STING pathway has undoubtedly expanded the potential for cancer treatment. While the effects of STING activation in combating cancer might vary, its ability to stimulate the immune response holds significant promise for novel therapeutic interventions. Therefore, further research is warranted to fully comprehend the diverse effects of STING activation in cancer. Particularly, the encouraging outcomes observed in using the cGAS-STING pathway for breast cancer treatment highlight its potential for further advancements. Consequently, targeting the STING protein represents a viable approach to potentially enhance future treatment outcomes in this context. This article summarized the regulation of STING and the influencing factors of cGAS-STING pathway. It further enriched the molecular mechanisms of breast cancer. It can provide some research ideas for the follow-up research on STING, and also provide experimental basis for clinical treatment of breast cancer.
